# Pollen holographic images and light-induced fluorescence measurements at the species level

**DOI:** 10.1038/s41597-025-05139-w

**Published:** 2025-05-13

**Authors:** Sophie Erb, Alexis Berne, Bernard Clot, Maria Lbadaoui-Darvas, Gian-Duri Lieberherr, Marie-Pierre Meurville, Fiona Tummon, Benoît Crouzy

**Affiliations:** 1 Federal Office of Meteorology and Climatology MeteoSwiss, 1530 Payerne, Switzerland; 2 Environmental Remote Sensing Laboratory (LTE), École Polytechnique Fédérale de Lausanne, 1015 Lausanne, Switzerland

**Keywords:** Environmental sciences, Public health, Plant sciences

## Abstract

The data collection presented here consists of holographic images and light-induced fluorescence measurements of pollen grains performed with the SwisensPoleno Jupiter, an instrument for real-time bioaerosol monitoring. This pollen collection, sampled directly from plants in their natural environment, is meant to provide reference datasets of airborne, mostly allergenic, pollen species found in central Europe. It has the advantage of including fluorescence measurements in addition to holographic images, giving an insight of both particle composition and morphology. Large reference datasets are necessary to develop automatic pollen monitoring tools but gathering fresh samples is complex, seasonally dependent and time consuming. Data sharing is the key to reduce data generation costs and improve identification of local and long-range transport pollen. These data are meant to be used as the basis for training new pollen identification algorithms that will make use of fluorescence measurements. Their potential reuse can also be to study pollen morphological and compositional variability at the species level.

## Background & Summary

One of the initial reasons to study airborne pollen was in response to a strong increase of allergies. Hence, monitoring was initiated by medical doctors themselves in the 20th century to diagnose and treat their patients’ symptoms. Pollen monitoring networks kept expanding worldwide^1^, following the increase of pollen allergy prevalence. After decades of manual measurements and thanks to new technologies, the automation of pollen monitoring started in the 2010s^2,3^.

Automatic pollen monitoring overcomes some of the major limitations of the manual methods, which include the low (daily) time resolution and the offset in data availability (being published up to a week later)^4^. This precluded the integration of pollen data into numerical weather models but has now been made possible thanks to the availability of real-time observations^5,6^. The use of recent innovative technologies^7^ has led to a paradigm change in the way pollen is measured. The integration of pollen data into numerical weather models improved pollen modelling quality and forecast. Additionally, thanks to the seamless provision of hourly data, information on current and forecasted pollen exposure is communicated more frequently to end-users. Several research groups have helped to develop automatic pollen monitoring over the past few years, with different instruments and species of interest being focused on. Since 2018, European efforts have been initiated to develop and standardise automatic pollen monitoring further, notably under the umbrella of the EUMETNET AutoPollen Programme^8^ and the EU Horizon Europe SYLVA project^9^. The development of standardised measurement methods is crucial to exchange pollen measurements between countries, particularly since pollen is transported across borders. While automatic operational networks start to emerge, the standardisation of measurement devices, and creation of standardised methods for evaluating the identification algorithms that they use, is still under development. The identification algorithms currently used are Machine Learning (ML) models such as convolutional neural networks or feature-based classifiers^10,11^, which are supervised ML methods. As such, they require labelled data that are complex, seasonally dependent and time consuming to produce: it requires botanical expertise, lab infrastructure and a dedicated instrument for producing datasets. Furthermore, there is currently no standardised protocol for sampling pollen or for generating pollen reference datasets.

The MeteoSwiss operational automatic pollen monitoring network (SwissPollen) is based on an airflow cytometer system that takes holographic images and light-induced fluorescence (LIF) measurements of single particles. Pollen identification is then performed by processing these measurements using a trained neural network. The standardisation of the LIF measurement made it possible to use it for particle identification. LIF measurements are complementary to holographic images in the sense that they provide information on the particle composition in addition to its appearance. Confusion between visually similar pollen taxa is then reduced^12^. Thus, creating a new large dataset including LIF, as opposed to the datasets used previously, which were based only on holography, was necessary to be able to train a new identification algorithm. Training a new model including LIF does not bring much complexity to the model as LIF data is a basic vector; but it is essential for eliminating artefacts such as false positive signals of particles that were misidentified based on holographic measurements. This gives further purpose to the present contribution which introduces a standard protocol for pollen sampling and dataset generation considering environmental as well as meteorological parameters.

The data presented here consist of holographic images and LIF measurements that were generated using the SwisensPoleno Jupiter^7,10,12^. This dataset is the largest homogeneous database of pollen holography and LIF measurements taken in natural environmental conditions. It covers 37 anemophilous plant species. All data from this database have been generated according to the previously mentioned standard protocol, ensuring homogeneity. The protocol is designed to be as comprehensive as possible while being simple to use, avoiding the need for specialised research laboratory equipment.

## Methods

### Measurement system

The SwisensPoleno Jupiter (Swisens AG) is an air flow cytometer that measures single airborne particles from 0.5–300 µm in size, with a sampling flow rate of 40 liters per minute. It concentrates the particles in the measurement chamber, where each particle triggers two lasers for size and velocity estimation by scattering. After particle triggering, two holographic images are taken simultaneously by cameras placed at 90° from each other and perpendicular to the airflow. Subsequently, the particle is excited by three laser sources (280, 365 and 405 nm) and the LIF signals are measured over five measurement channels (333–381, 411–459, 465–501, 539–585, and 658–694 nm) to which we refer by their central wavelength; i.e. 357, 435, 483, 562, and 676 nm, respectively.

To aerosolise bioaerosol particles in a controlled manner; i.e. at a stable rate with only chosen, known particles; we use a portable, custom-made device called the SwisensAtomizer (Swisens AG). This instrument relies on basic equipment and principles: particles are placed in a cuvette and are aerosolised through vibration and an air blower. The vibration frequency and amplitude can be controlled as well as the blower speed. The SwisensAtomizer is placed directly on top of the SwisensPoleno Jupiter so that the air pumped in by the instrument contains the pollen of interest. All the data measured can then be labelled at once with the corresponding particle name.

### Protocol

**Fig. 1:**
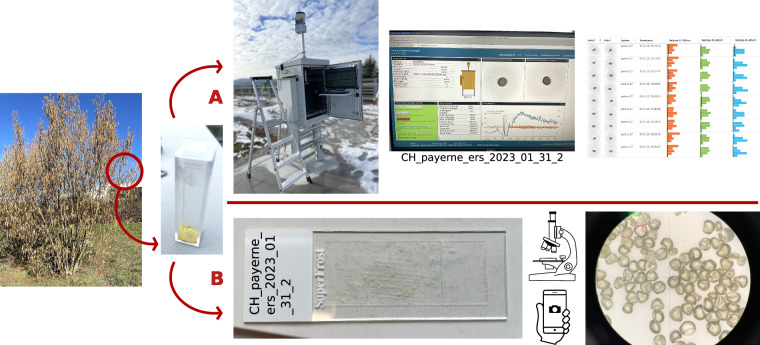
Data generation workflow. We start with the sample collection (left) and then proceed with (**A**) holography and LIF measurements with the SwisensPoleno Jupiter after aerosolisation with the SwisensAtomizer. In parallel (**B**) we examine the reference microscopy slide and take microscope images. The final data presented in this paper are on the right.

For this study, a dedicated protocol had to be designed to sample pollen and generate datasets using the SwisensPoleno Jupiter instrument. This protocol had to meet several expectations: a) be simple to use, b) be relevant for the whole pollen monitoring community, c) allow the collection of as much information on the data as possible. In this line, variables including location and environmental conditions as well as the parameterisation of the measurement instrument are taken together with microscopic images for every sample. This corresponds to a total of 43 variables per sample, which are listed and described in Table 1. We selected environmental variables, such as temperature, humidity and wind, that are known to have an effect on pollen release and morphology^13,14^. As the protocol is meant to be used by a large audience, we designed it so that people with no botanical expertise can also collect pollen from plants that they do not know. By taking pictures, identifications can be done later in the lab/office with access to appropriate literature and tools, and can also be verified by professional botanists (reducing identification bias) without disturbing the labelling of the sample. Since the sample name is the identification key of the sample, it should be as stable as possible to avoid renaming tubes, slides and datasets, potentially inducing errors. Note that from the database point of view, the sample name is the ID of the dataset so it should never change unless the dataset itself changes. For this database, plant identifications have been verified by Philippe Sauvain, botanical gardener, and Joëlle Magnin-Gonze, botanical curator from the Cantonal Museum of Natural Sciences in Lausanne, Switzerland. Latin scientific names from the International Plant Names Index^15^ were used to maximise compatibility with data from other locations. The protocol consists of two files: a .docx or .pdf form to store the metadata and an associated procedure .pdf file describing the actions to execute. They can be found on the GitHub repository^16^. It is the base methodology that we used for data collection and the corresponding workflow is illustrated in Fig. 1.

**Table 1:** List of metadata variables.

Variable	Type	Description
Dataset		
dataset_ID	integer	counting number associated to the dataset
dataset_date	date	dataset generation date in the format day.month.year
start_time, stop_time	time	starting, respectively stoping data generation time in Universal Time Coordinated (UTC) in the hh:mm:ss format
vibration_amplitude	integer	vibration amplitude that the SwisensAtomizer was set to during the stable phase of aerosolisation, given in percent relative to the aerosoliser max capacity
vibration_frequency	integer	vibration frequency during the stable phase of aerosolisation, given in Hz
blower_speed	integer	speed of the blower of the SwisensAtomizer as it was set during the stable phase of aerosolisation, given in percent relative to the aerosoliser max capacity
number_events	integer	number of particles measured in a dataset, one particle gives two holographic images and a LIF spectrum, which we refer to as one event
slide_picture	binary	yes or no, indicates whether a picture of the pollen grains under the microscope was taken or not
rh_aerosolisation	float	relative humiditiy measurements, when aerosolising and measuring the pollen sample, from the closest official MeteoSwiss station, using ventilated Thygan (Meteolabor AG) devices, two meters above ground, ensuring representativeness of the environment, in percent
temperature_aerosolisation	float	temperature measurements, when aerosolising and measuring the pollen sample, from the closest official MeteoSwiss station, using ventilated Thygan (Meteolabor AG) devices, two meters above ground, ensuring representativeness of the environment, in degrees Celsius
wind_aerosolisation	float	wind speed measurements, when aerosolising and measuring the pollen sample, from the closest official MeteoSwiss station, using ventilated Thygan (Meteolabor AG) devices, two meters above ground, ensuring representativeness of the environment, in kilometer per hour
wind_beaufort_aerosolisation	integer	wind index according to the Beaufort scale17, when aerosolising and measuring the pollen sample, does not need any measurement instrument and is an appreciation by the operator
comment	string	open field where any particular observation can be recorded, for exemple if the plant itself was collected for later pollen sampling
Sample		
sample_ID	integer	counting number associated to the sample
sample_name	string	composed as ZIPcode_location_initials_year_month_day_increment where ZIPcode is the ISO3166 international postal code standard, location is the plain name of the location, initials are the first two letters of the operator’s name and the first letter of their surname, year/month/day correspond to the sampling year/month/day, increment is a number starting at 1 and is incremented by one for each new sample collected by the same person at the same location and date
sample_date	date	sampling date in the format day.month.year
sampling_time	time	sampling time as Universal Time Coordinated (UTC) in the hh:mm format
closest_measurement_station	string	name of the closest meteorological station
temperature_sampling	float	temperature measurements, at the sampling time, from the closest official MeteoSwiss station, using ventilated Thygan (Meteolabor AG) devices, two meters above ground, ensuring representativeness of the environment, in degrees Celsius
rh_sampling	float	relative humiditiy measurements, at the sampling time, from the closest official MeteoSwiss station, using ventilated Thygan (Meteolabor AG) devices, two meters above ground, ensuring representativeness of the environment, in percent
wind_sampling	float	wind speed measurements, at the sampling time, from the closest official MeteoSwiss station, using ventilated Thygan (Meteolabor AG) devices, two meters above ground, ensuring representativeness of the environment, in kilmoeter per hour
wind_beaufort_sampling	integer	wind index according to the Beaufort scale^27^, at the sampling time, does not need any measurement instrument and is an appreciation by the operator
Dataset_link		
dataset_link_ID	integer	couting number matching the dataset_ID and the sample_ID
Plant		
plant_ID	integer	counting number associated to the plant
genus	string	plant genus according to the International Plant Names Index^15^
species	string	plant species according to the International Plant Names Index^15^
authorship	string	name of the person who named the plant species according to the International Plant Names Index^15^
latitude	float	WGS coordinates including five decimals
longitude	float	WGS coordinates including five decimals
group	binary	yes or no, indicates whether we have a group of several plant individuals, typically for herbaceous plants
plant_date	date	plant observation date
plant_picture	binary	yes or no, indicates whether a picture of the plant was taken or not
Poleno		
poleno_ID	string	name of the SwisensPoleno, composed of the letter P followed by the SwisensPoleno number
location	string	location of the SwisensPoleno
type	string	type of SwisensPoleno instrument, can be Mars, Neptune or Jupiter according to the manufacturer’s specifications
Atomizer		
atomizer_ID	string	name of the SwisensAtomizer, composed of the abbreviation of the the institution and a counting number
serial_number	string	serial number of the SwisensAtomizer
factory_date	date	date of manufacture of the SwisensAtomizer according to its label
Operator		
operator_ID	string	name or acronym of the person sampling
name	string	operator’s name
surname	string	operator’s surname
institution	string	name of the institution the operator works for

For each variable, its name, type and a brief description are provided. The list covers the metadata that are registered according to the sampling protocol.

There are several ways to collect pollen from plants: the standard method is to use a tube (ideal size is 50 ml) or a plastic box and shake it with the flower of the plant in the container so that the pollen falls into it. Depending on the species, it can be difficult to collect pollen directly and flowers have to be cut and brought back to the lab/office. Flowers are then laid on a sheet of paper for a few hours so that pollen falls on the sheet and can be collected afterwards in a tube. This flower sampling method was used for *Urtica dioica* (among others), whose male flowers explode, scattering pollen grains in the air^17^. If we had to use this alternative method, we mentioned the time lying on the paper sheet, including temperature and humidity conditions in the “comment” open field variable (see Table 1).

Aerosolising pollen grains with the SwisensAtomizer and measuring them with the SwisensPoleno Jupiter requires some experience with the instrument. Some general indications are given in the protocol files on the GitHub repository^16^. While pollen was being aerosolised and measured by the instrument; temperature, humidity, and wind speed were measured simultaneously a few meters away at the official MeteoSwiss weather station in Payerne. Therefore, the metadata contains temperature, humidity, and wind speed measurements both at the sampling and measuring time (see Table 1).

### Data cleaning

For training an identification algorithm, the selected data must contain only the particle type of interest. As we sample environmental data, the pollen sample can be contaminated with dust, other pollen grains, or plant debris; that are also aerosolised and measured. It is therefore necessary to clean the data, i.e. remove all particles that do not belong to the pollen type of interest. Using the SwisensDataExplorer graphical tool, MeteoSwiss pollen experts visually cleaned all datasets by manually deleting any particles that they did not recognise as the particle type of interest. This resulted in lists of IDs of particles to keep in order to obtain clean datasets, which we provide with the data. Typically, double particle images, particles with irregular shapes and particles outside the expected size range (too large or too small) were discarded. Nevertheless, blurred images were retained to ensure that the variability in the dataset is representative of what is seen with operational measurements. We want to stress that cleaned versions of the datasets are not ground truth and it is very unlikely that two people cleaning the same dataset will end up with exactly the same clean version. However, by following the guidelines provided above, we ensure that the essential features of the dataset are preserved.

## Data Records

The data presented in this paper were generated between January 2023 and May 2024, and are composed of 59 datasets, as listed in Table 2. Each dataset corresponds to a sample which contains pollen either from a single individual plant or several individuals of the same species. This is indicated in the sample metadata in the binary “group” variable (“yes” meaning several individuals, see Table 1). As shown in Table 2, data records cover a total of 37 different species, with several samples for some species. The sampling design allows one to take advantage of opportunities: every anemophilous plant releasing pollen at the collection time was sampled. However, we tried to have as many allergenic species as possible, focusing on the most important ones. Additionally, we aimed to sample at the species or even individual plant level to allow deeper investigation of morphological variability. As the goal was to collect the largest panel of pollen possible based on opportunities, some samples were of bad quality or produced only a small number of particles as a result of the stickiness of the pollen grains or late season sampling (few or shrivelled pollen grains). For example, the dataset for *Acer pseudoplatanus* contains only 457 particles because the pollen grains are sticky and aerosolise very poorly. This is expected as *Acer* trees are mainly pollinated by insects. Similar stickiness issues were encountered also for *Carex flacca*, *Tilia cordata* and *Castanea sativa*. *Ostrya carpinifolia* on the other hand was at the end of its flowering season and collected after a week of sunny, warm and windy days. The flowering stage of the plant plays an important role in the quality of the sample. At the beginning of the season, plants release the first pollen grains, usually in low to medium concentrations, which may be at different stages of maturity as some grains are still maturing. At the peak of the flowering season, mainly mature pollen is released in relatively large quantities; but by the end of the season, few pollen grains remain on the plant and may be deformed and/or dry. We usually try to collect pollen during the main flowering period, when pollen is abundant and well formed, to get the best representativeness.

**Table 2:** List of datasets in the data collection.

	Sample_name	Genus	Species	Authorship	Complete_events	Incomplete_events
1	CH_avenches_ers_2023_03_02_1	*Corylus*	*avellana var. contorta*	Bean	33615	2355
2	CH_avenches_ers_2023_03_15_1	*Corylus*	*avellana var. contorta*	Bean	24231	1301
3	CH_avenches_ers_2023_03_17_1	*Corylus*	*avellana var. contorta*	Bean	19132	939
4	CH_avenches_ers_2023_03_17_2	*Corylus*	*avellana var. contorta*	Bean	17785	637
5	CH_avenches_ers_2023_04_06_1	*Carpinus*	*betulus*	L.	15495	2029
6	CH_avenches_ers_2023_04_06_2	*Carpinus*	*betulus*	L.	16365	2710
7	CH_avenches_ers_2023_04_06_3	*Betula*	*pendula*	Roth	23671	2105
8	CH_avenches_ers_2023_04_18_1	*Betula*	*pendula*	Roth	18419	897
9	CH_avenches_ers_2023_04_27_1	*Betula*	*pendula*	Roth	31472	1975
10	CH_avenches_ers_2023_04_27_2	*Betula*	*pendula*	Roth	19968	1002
11	CH_avenches_ers_2023_04_27_3	*Pseudotsuga*	*menziesii var. glauca*	(Mayr) Franco	15240	1760
12	CH_avenches_ers_2023_05_05_1	*Abies*	*bornmuelleriana*	Mattf.	12423	5536
13	CH_avenches_ers_2023_05_05_2	*Liquidambar*	*styraciflua*	L.	15210	4568
14	CH_bellinzona_ers_2023_05_11_1	*Trachycarpus*	*fortunei*	(Hook.) H.Wendl.	19664	1407
15	CH_boveresse_ers_2023_03_12_1	*Corylus*	*avellana*	L.	15908	1071
16	CH_boveresse_ers_2023_03_12_3	*Corylus*	*avellana*	L.	19149	625
17	CH_boveresse_ers_2023_05_06_1	*Acer*	*pseudoplatanus*	L.	352	105
18	CH_boveresse_ers_2023_05_06_2	*Sambucus*	*racemosa*	L.	19362	616
19	CH_boveresse_ers_2023_05_07_1	*Alopecurus*	*pratensis*	L.	16383	6395
20	CH_boveresse_ers_2023_05_07_2	*Plantago*	*lanceolata*	L.	18756	3050
21	CH_boveresse_ers_2023_05_07_3	*Carex*	*flacca*	Schreb.	16131	6687
22	CH_boveresse_ers_2023_05_13_1	*Picea*	*abies*	(L.) H.Karst.	16723	4656
23	CH_boveresse_ers_2023_08_01_1	*Artemisia*	*absinthium*	L.	17766	1061
24	CH_chevroux_ers_2023_02_21_1	*Alnus*	*glutinosa*	(L.) Gaertn.	12275	1014
25	CH_chevroux_ers_2023_02_21_2	*Alnus*	*glutinosa*	(L.) Gaertn.	34124	1106
26	CH_chevroux_lax_2023_02_21_1	*Corylus*	*avellana*	L.	16429	784
27	CH_chevroux_lax_2023_02_21_2	*Corylus*	*avellana*	L.	15995	569
28	CH_chevroux_lax_2023_02_21_3	*Alnus*	*glutinosa*	(L.) Gaertn.	31124	839
29	CH_fribourg_tuf_2023_10_30_1	*Cedrus*	*atlantica*	(Endl.) G.Manetti ex Carrière	15155	2064
30	CH_lausanne_ers_2023_09_11_4	*Ambrosia*	*artemisiifolia*	L.	20681	1129
31	CH_morat_ers_2024_04_23_1	*Quercus*	*robur*	L.	32920	3170
32	CH_neuchatel_ers_2023_05_12_1	*Pinus*	*sylvestris*	Lour.	15140	743
33	CH_payerne_clb_2023_05_05_1	*Rumex*	*obtusifolius*	L.	19238	1032
34	CH_payerne_ers_2023_01_31_1	*Corylus*	*avellana*	L.	11711	769
35	CH_payerne_ers_2023_01_31_2	*Corylus*	*avellana*	L.	23190	1333
36	CH_payerne_ers_2023_02_13_1	*Alnus*	*incana*	(L.) Moench	11572	488
37	CH_payerne_ers_2023_02_13_2	*Corylus*	*avellana*	L.	13566	633
38	CH_payerne_ers_2023_03_20_1	*Larix*	*decidua*	Mill.	15484	2082
39	CH_payerne_ers_2023_05_04_1	*Platanus*	*hispanica*	Münchh.	17681	1000
40	CH_payerne_ers_2023_05_04_2	*Fagus*	*sylvatica*	L.	5298	1504
41	CH_payerne_ers_2023_05_16_1	*Bromus*	*sterilis*	L.	15724	8212
42	CH_payerne_ers_2023_05_16_2	*Lolium*	*multiflorum*	Gaudin	16214	6269
43	CH_payerne_ers_2023_05_30_1	*Dactylis*	*glomerata*	L.	15311	7574
44	CH_payerne_ers_2023_05_30_2	*Trisetum*	*flavescens*	(L.) P.Beauv.	19100	4857
45	CH_payerne_ers_2023_06_19_1	*Castanea*	*sativa*	Mill.	4595	1208
46	CH_payerne_ers_2023_06_20_1	*Tilia*	*cordata*	Mill.	282	199
47	CH_payerne_ers_2023_08_02_1	*Urtica*	*dioica*	L.	25781	2835
48	CH_payerne_ers_2023_08_14_1	*Trisetum*	*flavescens*	(L.) P.Beauv.	23529	8627
49	CH_payerne_ers_2023_10_03_1	*Cedrus*	*atlantica*	(Endl.) G.Manetti ex Carrière	11261	1413
50	CH_payerne_ers_2024_04_23_1	*Quercus*	*robur*	L.	12784	1607
51	CH_payerne_ers_2024_05_03_4	*Juglans*	*regia*	L.	13548	1309
52	CH_payerne_ers_2024_05_03_5	*Juglans*	*regia*	L.	15805	1463
53	CH_payerne_lax_2023_02_23_1	*Cryptomeria*	*japonica*	(L.f.) D.Don	17417	1114
54	CH_tremona_ers_2024_04_16_5	*Ostrya*	*carpinifolia*	Scop.	472	24
55	CH_villars-sur-glane_ers_2023_02_28_1	*Taxus*	*baccata*	Thunb.	61301	1971
56	CH_villars-sur-glane_ers_2023_03_14_1	*Corylus*	*avellana var. contorta*	Bean	17389	676
57	FR_labege_ers_2023_04_23_1	*Fraxinus*	*ornus*	L.	19367	480
58	FR_saint-jean-de-la-porte_ers_2023_04_16_1	*Plantago*	*lanceolata*	L.	16463	1665
59	FR_saint-jean-de-la-porte_ers_2023_04_16_2	*Carex*	*flacca*	Schreb.	43	6

The sample names, sorted by alphabetical order, correspond to the dataset names; genus, species and authorship compose the full scientific name of each species; complete versus incomplete events are the number of events (particle measurements) with two holographic images (non null maximum intensity value) and LIF spectrum versus the number of events that have at least one empty image or missing LIF spectrum.

**Fig. 2:**
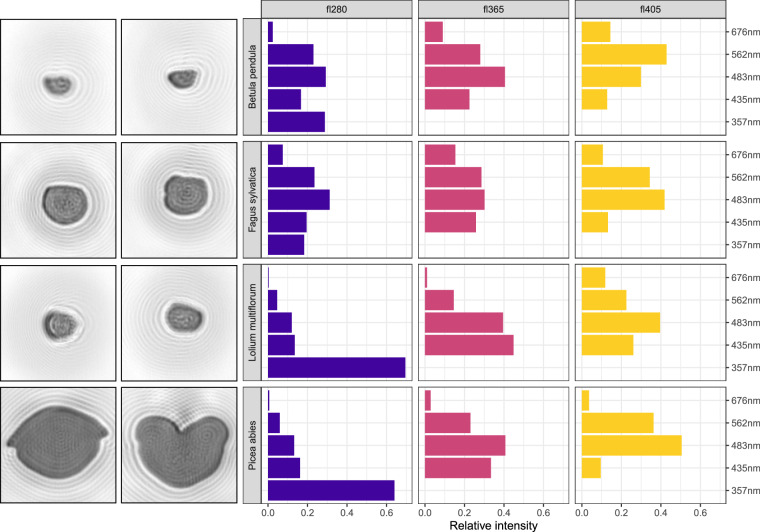
Examples of measurement events of pollen grains of four different species. *Betula pendula*, *Fagus sylvatica*, *Lolium multiflorum* and *Picea abies* were chosen to represent the large variability in the data. Each row shows the two holographic images and the relative LIF spectrum (normalised to 1) of one pollen grain separated by excitation source.

All particles were measured using a SwisensPoleno Jupiter. This means that for each particle two holographic images and one LIF excitation-emission matrix were recorded. Figure 2 shows examples of measurements from four different species. Most of the samples were sourced in the area surrounding the MeteoSwiss regional Centre in Payerne, Switzerland, but a number of them come from France. We produced all the datasets under outdoor conditions, on the roof of the MeteoSwiss station, with a redundant SwisensPoleno Jupiter that is permanently installed there as part of the SwissPollen network. The instrument itself was changed for maintenance twice; but the configuration remained the same.

All the data and metadata are stored as a single.tar archive hosted on a Zenodo^18^ repository with a fixed DOI (10.5281/zenodo.13991959)^19^. Since the holographic images are greyscale and of limited resolution (200 by 200 pixels), we also took microscopic images of each sample for a more accurate representation of the pollen grains. For each sample, we also photographed the plant or individuals sampled to ensure correct identification of each species and to obtain indices on the environmental conditions and the state of each individual plant. Photos include a general picture of the whole plant and its environment and then closer images showing details such as leaves, flowers, and the trunk or the base of the plant for identification purposes. All these images and observations are available in the data repository. It is important to note that some species, especially tree species, cannot be accurately identified at flowering time due to the absence of mature leaves and fruits. It is sometimes necessary to return a few months later to photograph and/or collect some material to make or verify an accurate identification. For example, at the collection time, the two *Quercus robur* in this data collection could only be assigned to the genus *Quercus* as they had no leaves nor fruits. We accurately identified the species about six months later as we collected mature leaves and fruits. For species of the Poaceae family, we highly recommend plucking one individual plant from root to tip as some identification criteria can only be observed with a magnifying glass.

The repository contains two .tar archives: *MCH_2023-2024_datasets_pollen.tar*, containing all the data, and *MCH_2023-2024_datasets_pollen_sub.tar*, a subsample of the data to facilitate exploration of its structure. The subsample contains the four datasets corresponding to Fig. 2. Each archive contains three subfolders: (1) *plant_pictures_and_microscope*, (2) *data_zip_files* and (3) *data_clean_ids* and three single files: a metadata .xlsx file (*db_metadata_pollen_2023-2024.xlsx*), a .json descriptor file (*JSONfile_documentation.json*) and a .txt *README* summary file. The three subfolders contain (1) photos of the plants from which samples were collected as well as microscopic images of its pollen, (2) SwisensPoleno Jupiter measurements as .zip files, and (3) lists of IDs (Universally Unique IDentifiers (*UUID)* and corresponding *event_ID*) of particles to keep to obtain a clean dataset. There are 59 samples in each subfolder.

Particles for which there were no images were removed from the final dataset provided in the *data_zip_files* subfolder of the repository. The reason for this is that empty images are usually due to measurement issues (e.g. saturation artifacts) which are inherent to measurement campaigns; but do not reflect what is observed operationally. Also the fluorescence measurements in these cases are biased and therefore such particles are of no use for training identification algorithms. Finally, the lists of IDs provided allow the obtainment of a clean version of each dataset, ready for algorithm training, corresponding to the one used to train the new 2025 MeteoSwiss operational model. Clean versions of the datasets can be obtained by filtering the .zip files according to the .txt lists of IDs. We provide a clean version of the data so that it can be used directly to train new models, but we also strongly encourage developments to allow automatic cleaning of these data to avoid/limit human bias, to reduce workload and to ensure reproducibility.

**Fig. 3:**
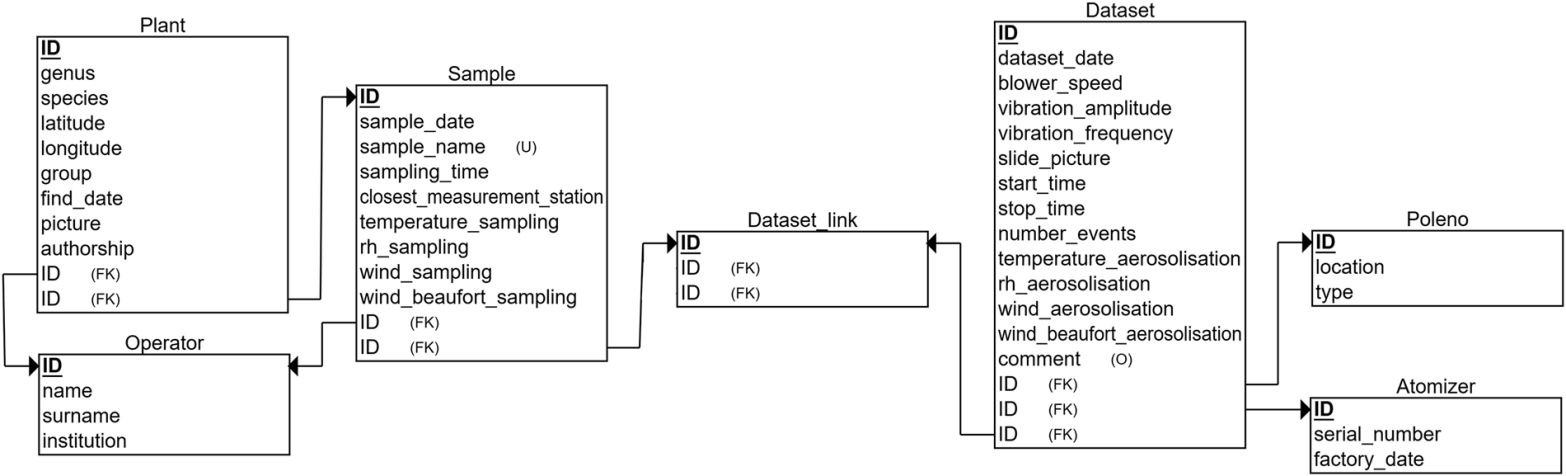
Relational schema of the metadata database. For each sample, all the metadata displayed here is saved. The metadata is contained in an .xlsx file. Each box corresponds to a separate .xlsx sheet; arrows between boxes show the relations between the sheets; variables listed in each box correspond to the columns in the sheet. Some variables have special tags: (FK) means foreign key, i.e. a variable that comes from another sheet, (U) means unique such that every value for this variable is different from the others and (O) means optional for variables that do not necessarily contain a value.

The data from the SwisensPoleno Jupiter given in the .zip files always have the same structure. For each particle, also called an “event”, there are two .png images and one .json file. Filenames are unique and contain information about the measurement instrument, dataset creation date, event ID, and type of measurement (image 0, 1 or .json). For example: *poleno-27_2023-03-03_09.21.59.729780_ev.computed_data.holography.image_pairs.0.0.rec_mag.png* is a particle measured by the SwisensPoleno Jupiter n° 27 on 3 March 2023, with event ID n° 09.21.59.729780 and is the image 0. Each .json file contains measurement information including the image features and the LIF measurements. A descriptive .json file, provided by the manufacturer and containing all the variables that can be registered by the SwisensPoleno Jupiter is provided in the Zenodo archive. In this reference .json file, the tags *_href* and *_doc* provide information about the path and the description of each variable in the .json file, respectively. Note that the .json files provided with the data may not contain as many fields as the .json descriptor file due to the coexistence of different SwisensPoleno software versions and configurations. The .xlsx metadata file (*db_metadata_pollen_2023-2024.xlsx*) contains all the information and environmental data for each of the 59 samples. Figure 3 shows the relational schema of this metadata database, that corresponds to the variables listed in Table 1. Finally, a *README.txt* file summarises the content of the archive.

## Technical Validation

**Fig. 4:**
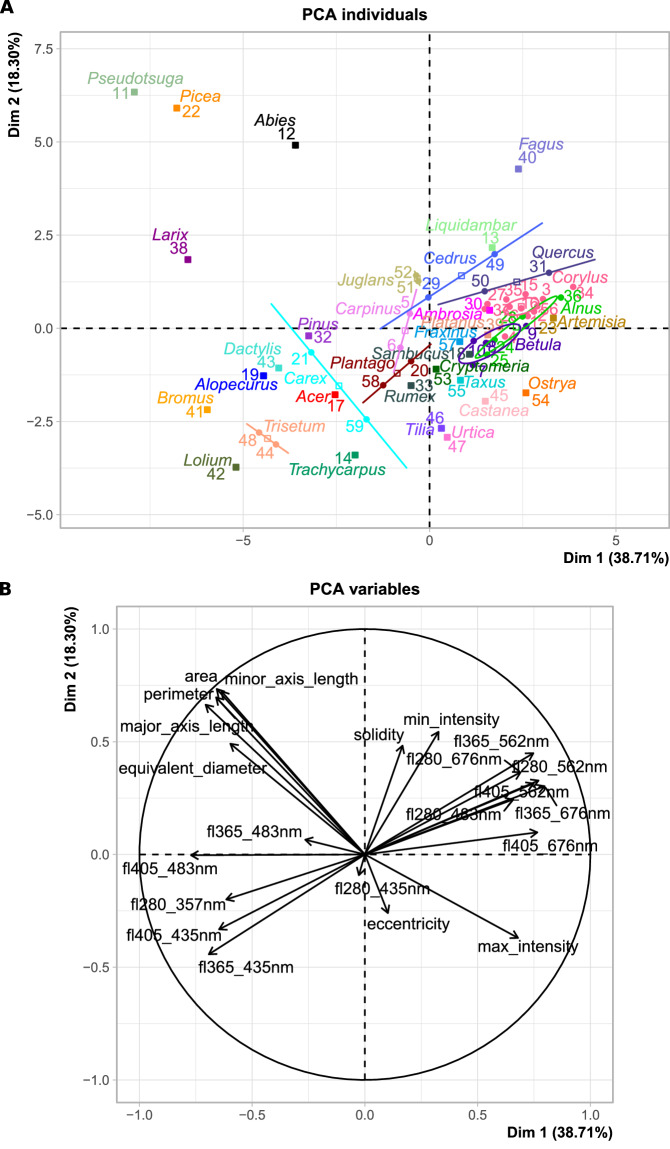
PCA graphs of (**A**) the individuals and (**B**) the variables. Both graphs can be superposed to see which variables are key features of which dataset. In (**A**) one dot corresponds to one dataset, coloured by genus and grouped by a standard ellipse (a line if only two points). Datasets that are further away are considered more different than closer ones. In (**B**) the length of each arrow shows the weight of each variable for separating datasets. Percentages of Dim 1 and 2 represent the variability of the data that can be explained by those axes.

We verified the quality of the data described here by comparison with published data. We compared the size and shape of each pollen species with the online free palynological database (PalDat)^20^ and LIF patterns with the measurements from Pöhlker *et al*.^21^. For example, the size of *Corylus avellana* on PalDat is indicated as medium-sized, i.e., 26–50 µm, and the median major and minor axes for our seven *C. avellana* datasets are in the range 44–52 and 35–41 µm, respectively. Similarly for LIF, the intensity patterns in excitation-emission matrices for *Betula* and *Lolium* in Pöhlker *et al*., 2013 (Fig. 4) is coherent with what we show in Fig. 2. *Betula*’s higher LIF intensity is with the 405 nm laser source in the 562 nm channel corresponding to the maximum intensity in the reference article (450–550 nm source and 450–600 nm emission). Similarly, *Lolium* exhibits higher intensities with all three laser sources (280, 365 and 405 nm) in the channels 357, 435 and 483 nm. In Pöhlker *et al*., 2013, for any source (ranging from 280–500 nm) high intensities are measured in the range 400–550 nm.

We explored the consistency of the measurements using a Principal Component Analysis (PCA) on the median of each image feature and LIF measurement (extracted from the .json files) for all samples. We chose this method for its simplicity and interpretability. Results were obtained using the *PCA* function from the “FactoMineR” R package^22^, version 2.11. The median aggregation was necessary since taking all particles separately was computationally too heavy and unreadable (more than 1 million points). Although the data were transformed to median variables, in essence, the assumptions for the PCA were respected (59 observations for 25 variables with monotone relationships). The median aggregation is acceptable since the goal of the PCA presented here is not to quantify variability or cluster groups but rather to observe whether pollen species express well their known characteristics. Figure 4A shows the position of each dataset (coloured by genus) relative to others based on morphological and compositional information. Figure 4B displays the variables driving group separation. The two sub-figures are to be interpreted in parallel, i.e. the position of a dataset in 4A is linked to the variables in 4B. The position of variables and length of their respective arrows on Fig. 4B shows how important variables are to distinguish the corresponding dataset in Fig. 4A. Datasets in the top left quadrant are well characterised by their morphological size (particle perimeter, minor/major axis, area, equivalent diameter) while in the top right quadrant, the key characteristics are related to the high LIF intensities in higher emission wavelength channels (565 and 676 nm). In the bottom left quadrant, datasets typically have distinctive LIF spectra with high LIF intensities in lower emission wavelength channels (357 and 435 nm). Finally, datasets in the bottom right quadrant have high pixel intensity with eccentric shapes.

The variable graph, Fig. 4B, also reveals that most image features are perpendicular to LIF, indicating that morphology and LIF measurements are not related. This underlines the quality of the data and the added value of LIF measurements to complete holography in the characterization of airborne pollen grains as previously shown in Erb *et al*.^12^.

Finally, considering the position of each sample in the individual graph, Fig. 4A, together with the variables, Fig. 4B, we can verify if they match known particle features. From the literature, we know that pollen grains from trees of the Pinaceae family are large with air sacs giving them a typical shape (see Fig. 2, row 4). On Fig. 4A, they are placed separately from other pollen datasets, in the top left quadrant characterised by large sizes. On the other hand, grass pollen exhibits a characteristic LIF spectrum of high intensity^21^ and we observe the same distinction of this group with *Alopecurus, Bromus, Dactylis*, *Lolium* and *Trisetum* together in the bottom left quadrant driven by high LIF intensities at emission wavelengths 357 and 435 nm.

## Usage Notes

To work with the data, one should first download the .tar archive from Zenodo. The dataset user can train a new particle identification model based on the content of the .zip files. As all the measurements (.jpg and .json) contain the particle ID in their name, and lists of IDs for clean datasets are provided, one can filter the data as they read it using a programming language like R^23^ or Python^24^. Ideally, one would try filtering the data themselves automatically so as to reduce any potential bias as well as labour time. This can be done by directly filtering the images based on the .json data. The plant and pollen microscopic images are meant to help the cleaning step as one can compare the content of the sample to what is measured by the SwisensPoleno Jupiter. Finally, the metadata database is easy to read with a programming language (for example, R with the *read_excel* function from the “readxl”^25^ package or Python with the *read_excel* function of the “pandas”^26^ package) and provides information on environmental variables but also on the sample itself. It can be useful to load it so that one can match the sample names to the species. We hope that this data will be used to investigate automatic data cleaning methods, develop different pollen identification models, and test various architectures and methods.

## Data Availability

The code to load, format and analyse the data (via PCA) is available on GitHub^16^. It uses the programming language R^23^, version 4.3.1.
